# Estimated diagnostic performance of prostate MRI performed with clinical suspicion of prostate cancer

**DOI:** 10.1186/s13244-024-01845-y

**Published:** 2024-11-15

**Authors:** Hirotsugu Nakai, Hiroaki Takahashi, Jordan D. LeGout, Akira Kawashima, Adam T. Froemming, Derek J. Lomas, Mitchell R. Humphreys, Chandler Dora, Naoki Takahashi

**Affiliations:** 1 Department of Radiology, Mayo Clinic, Rochester, MN US; 2 Department of Radiology, Mayo Clinic, Jacksonville, FL US; 3 Department of Radiology, Mayo Clinic, Scottsdale, AZ US; 4 Department of Urology, Mayo Clinic, Rochester, MN US; 5 Department of Urology, Mayo Clinic, Scottsdale, AZ US; 6 Department of Urology, Mayo Clinic, Jacksonville, FL US

**Keywords:** Magnetic resonance imaging, Prostatic neoplasms, Early detection of cancer, Models (statistical)

## Abstract

**Purpose:**

To assess the diagnostic performance of prostate MRI by estimating the proportion of clinically significant prostate cancer (csPCa) in patients without prostate pathology.

**Materials and methods:**

This three-center retrospective study included prostate MRI examinations performed for clinical suspicion of csPCa (Grade group ≥ 2) between 2018 and 2022. Examinations were divided into two groups: pathological diagnosis within 1 year after the MRI (post-MRI pathology) is present and absent. Risk prediction models were developed using the extracted eleven common predictive variables from the patients with post-MRI pathology. Then, the csPCa proportion in the patients without post-MRI pathology was estimated by applying the model. The area under the receiver operating characteristic curve (AUC), sensitivity, specificity, and positive and negative predictive values (PPV/NPV) of prostate MRI in diagnosing csPCa were subsequently calculated for patients with and without post-MRI prostate pathology (estimated statistics) with a positive threshold of PI-RADS ≥ 3.

**Results:**

Of 12,191 examinations enrolled (mean age, 65.7 years ± 8.4 [standard deviation]), PI-RADS 1–2 was most frequently assigned (55.4%) with the lowest pathological confirmation rate of 14.0–18.2%. Post-MRI prostate pathology was found in 5670 (46.5%) examinations. The estimated csPCa proportions across facilities were 12.6–15.3%, 18.4–31.4%, 45.7–69.9%, and 75.4–88.3% in PI-RADS scores of 1–2, 3, 4, and 5, respectively. The estimated (observed) performance statistics were as follows: AUC, 0.78–0.81 (0.76–0.79); sensitivity, 76.6–77.3%; specificity, 67.5–78.6%; PPV, 49.8–66.6% (52.0–67.7%); and NPV, 84.4–87.2% (82.4–86.6%).

**Conclusion:**

We proposed a method to estimate the probabilities harboring csPCa for patients who underwent prostate MRI examinations, which allows us to understand the PI-RADS diagnostic performance with several metrics.

**Clinical relevance statement:**

The reported estimated performance metrics are expected to aid in understanding the true diagnostic value of PI-RADS in the entire prostate MRI population performed with clinical suspicion of prostate cancer.

**Key Points:**

- Calculating performance metrics only from patients who underwent prostate biopsy may be biased due to biopsy selection criteria, especially in PI-RADS 1–2.
- The estimated area under the receiver operating characteristic curve of PI-RADS in the entire prostate MRI population ranged from 0.78 to 0.81 at three facilities.
- The estimated statistics are expected to help us understand the true PI-RADS performance and serve as a reference for future studies.

**Graphical Abstract:**

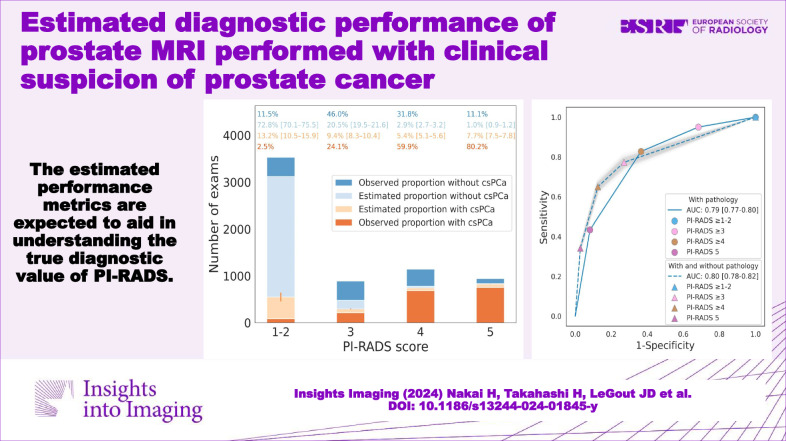

## Introduction

The Prostate Imaging-Reporting and Data System (PI-RADS) is designed to improve early diagnosis of clinically significant prostate cancer (csPCa) [1]. Positive predictive value (PPV) is the most widely used performance metric for PI-RADS positive cases, commonly defined as PI-RADS ≥ 3 [2–5]. As using a single performance metric (e.g., PPV alone) can be misleading [6], multiple metrics are needed to assess the diagnostic performance of prostate MRI. Recently, cancer detection rate (CDR) and abnormal interpretation rate (AIR) have been proposed as new combined metrics for PI-RADS-positive cases [7, 8].

Similarly, negative predictive value (NPV) is a common performance metric for PI-RADS negative cases, commonly defined as PI-RADS 1–2 [9, 10]. NPV influences clinicians’ decisions to avoid prostate biopsy [11], but similarly, using NPV alone can be misleading as a performance metric. Unfortunately, there are no other well-established metrics for PI-RADS negative cases.

Calculating performance metrics only from patients who underwent prostate biopsy may be biased due to biopsy selection criteria. This concern is especially true for PI-RADS negative cases due to a low prostate biopsy rate, for example, 12.4% in a previous study [12].

In this study, we propose to estimate diagnostic performance metrics from the entire patients undergoing prostate MRI for clinical suspicion of csPCa. For this, the csPCa proportion in patients without pathological confirmation needs to be estimated. To make this estimation reasonable, several known csPCa-associated factors should be taken into account, including age [13], prostate-specific antigen density (PSAD) [14, 15], previous history of prostate biopsy [16], and family history of prostate cancer [17]. We recently developed a natural language processing pipeline to capture such information from radiology reports and clinical notes [18]. By estimating the csPCa proportion, we can calculate not only NPV but also other key performance metrics, including sensitivity, specificity, and area under the receiver operating characteristic curve (AUC). This may help us understand the true diagnostic performance of prostate MRI regardless of the presence of pathological confirmation of the prostate.

The purpose of this study was to estimate the diagnostic performance of prostate MRI performed for clinical suspicion of csPCa by estimating the csPCa proportion in patients without pathological confirmation of the prostate.

## Materials and methods

This HIPAA-compliant retrospective study was approved by our institutional review board with an informed consent waiver. Thousands of patients overlapped with published works regarding CDR of prostate MRI [7, 8, 18–20].

### Study population

**Fig. 1:**
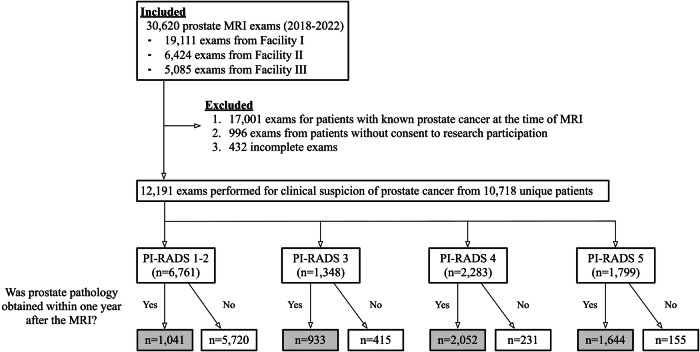
Patient flowchart. PI-RADS, Prostate Imaging-Reporting and Data System

Patients who underwent prostate MRI at three facilities of a single institution from 2018 to 2022 were included. Patients who had known prostate cancer (Grade group ≥ 1) at the time of MRI or had incomplete examinations were excluded (Fig. 1).

### MRI

All examinations were performed under PI-RADS version 2.0 or 2.1 technical specifications. Most studies were performed on 3-T scanners (GE Medical Systems, Illinois, U.S., or Siemens Healthineers, Erlangen, Germany), but 1.5-T scanners were used when the 3-T magnet was contraindicated. Contrast material was used unless contraindicated. Board-certified, fellowship-trained abdominal radiologists interpreted the MRI based on PI-RADS criteria using the same standardized report template. Prostate segmentation and 3-dimensional lesion markings were made on DynaCAD (Philips Healthcare, Best, the Netherlands) for a targeted biopsy when PI-RADS ≥ 3 lesions existed.

### Prostate biopsy

Trans-rectal or trans-perineal ultrasound-guided targeted biopsy (3–5 cores per lesion) was performed by urologists utilizing fusion software (UroNav, Philips Healthcare). Systematic biopsies were performed simultaneously (10–12 cores). In PI-RADS 1–2, systematic biopsies were performed if clinical suspicion of csPCa was high.

### Estimation of csPCa proportion

In overview, the csPCa proportion in patients who did not have pathological confirmation within 1 year after the MRI was estimated using logistic regression models developed from patients who had pathological confirmation for each PI-RADS score.

#### Data collection

The target variable was the presence of csPCa within 1 year after the MRI, which was extracted from pathology reports between January 2018 and October 2023. The csPCa was defined as Grade group ≥ 2 or prostate cancer pathologically diagnosed only from the metastatic foci without a Gleason score. The following ten predictive variables at the time of MRI were extracted from clinical notes or MRI reports: age; facility; prostate-specific antigen (PSA) values at the time of MRI; presence of previous benign prostate biopsy [16]; patient-level highest PI-RADS score; prostate volume measured on MRI; family history of prostate cancer; family history of breast cancer (alternative of possible BRCA gene mutation [17, 21]); presence of prostate nodule on digital rectal exam [22]; and race [13]. Additionally, the PSAD value was calculated by dividing the PSA value by prostate volume.

To extract clinical notes, an in-house software, MedTagger (https://github.com/OHNLP/MedTagger/), [23] was used. Then, patient-level categorization was performed by applying a developed natural language processing pipeline using Bidirectional Encoder Representations from Transformers [18].

#### Data preprocessing and feature selection

First, PSA and prostate volume were log-transformed to make the originally skewed distribution more normal. Second, continuous variables, including those two and age, were standardized to have means of 0 and standard deviations of 1. Facility information, a categorical variable with three classes, was binarized using one-hot encoding. Third, missing value imputations were performed using multiple imputations by chained equations technique [24] for variables with missing values of transformed PSA and transformed prostate volume. Then, PSAD was calculated, log-transformed, and standardized. Features to be included in the logistic regression model were selected using a subset of data with a forward feature selection technique. The most parsimonious set of features with low error was chosen using the area under the receiver operating characteristic curves and the one standard error rule [25]. The final included features were age, previous history of benign prostate biopsy, facility, PI-RADS score, PSAD, and prostate volume. The details of those steps will be reported separately.

#### Bootstrap aggregation

**Fig. 2:**
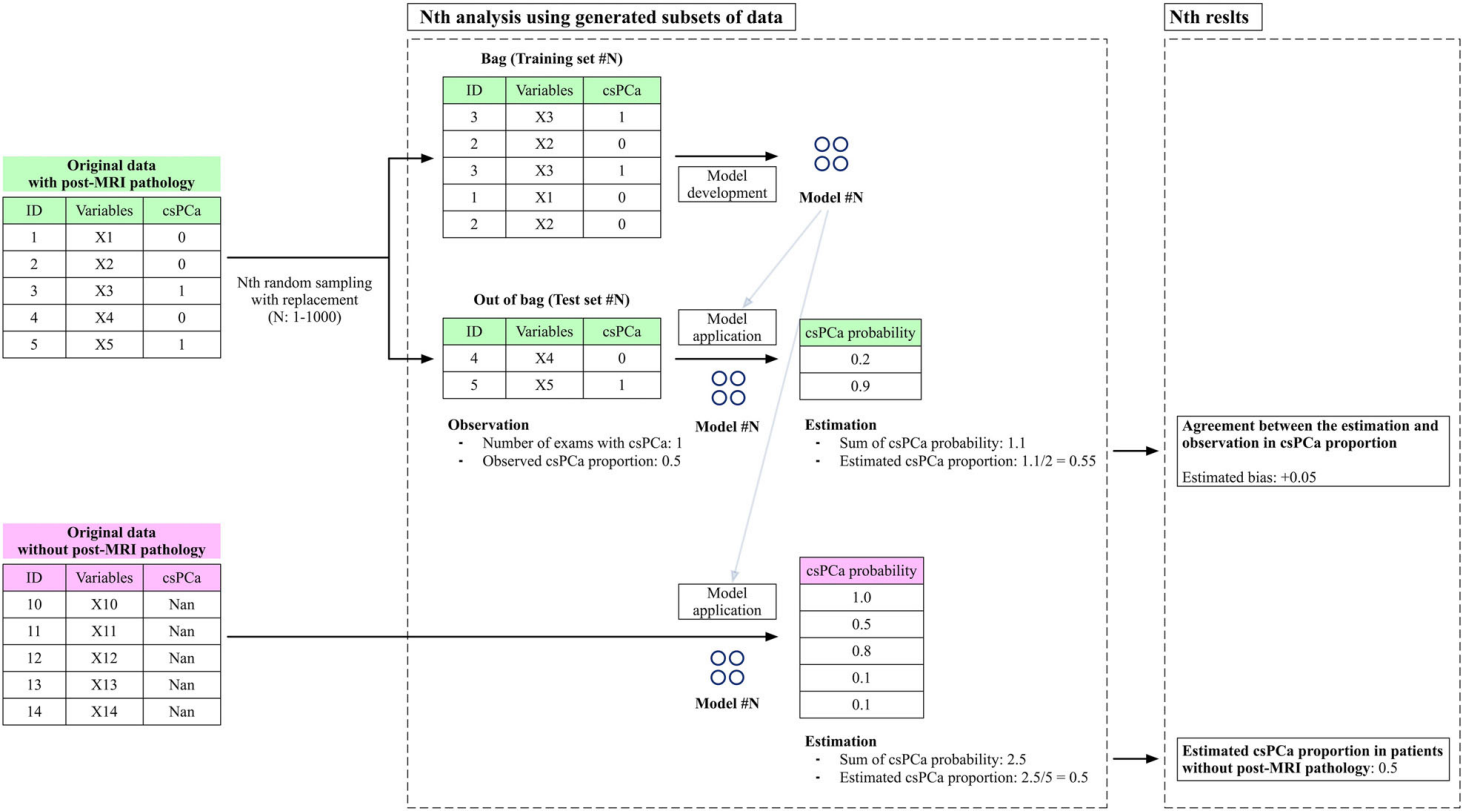
The schema of estimating the proportion of clinically significant prostate cancer in patients without pathological confirmation. The proportion of clinically significant prostate cancer (csPCa) in patients without pathological confirmation was estimated through a thousand repeated processes called bootstrap aggregating, a commonly used machine learning technique for reducing variance. For each repetition, a model was independently created using a subset of the dataset called “bag”, which was chosen by sampling with replacement. The remaining data not chosen in the sampling process was called “out of bag” and used for calculating the degree of estimation bias in the population with pathological confirmation. A model consisted of three calibrated logistic models created through threefold cross-validation and outputted the average of their outputs. In each fold, a logistic regression model was developed using two-thirds of the “bag”. Then, its prediction on the remaining subset was used to fit the agreement to the observed csPCa proportion through a sigmoid regressor. The thousand estimated results were aggregated to calculate the mean with the 95% confidence interval using the percentiles of the bootstrap distribution. This process was performed separately for data per PI-RADS score

A thousand prediction models were created using different subsets of data generated by random sampling with replacement [26] from each PI-RADS population with pathological confirmation. Then, the models were applied to patient data without pathological confirmation to estimate the csPCa proportion, defined as the average of the model’s outputs of all patients at each PI-RADS score. Similarly, the degree of estimation bias, a difference between observed and estimated csPCa proportions, was calculated using the population with pathological confirmation but not selected at random sampling (out of bag). Further details regarding this process can be found in Fig. 2.

The above data preprocessing step was independently performed for each bootstrap repetition to avoid data leakage. Standardization was based on training sets and applied to other data. The average of bootstrap statistics was taken to compute a more accurate estimate with degrees of uncertainty.

### Analyses

The primary analysis estimated the PI-RADS score-level csPCa proportion in patients who did not have pathological confirmation of the prostate within 1 year after the MRI. Then, the AUC and the following statistics were calculated:

$${Sensitivity}=\frac{{estimated}\, {{\#}}\, \left(\ge {PI}-{RADS\; i}\cap {csPCa}+\right)}{{{\, \#\,}}({csPCa}+)}$$

$${Specificity}=\frac{{estimated}\, \, {{\#}}\, \, \left( < \, {PI}-{RADS\; i}\cap {csPCa}-\right)}{{estimated}\,\,({{\#}}\, {csPCa}-)}$$

$${estimated}\, \left({observed}\right) \, {PPV}=\frac{{estimated} \, \left({observed}\right){{\, \#}}\, \left(\ge {PI}-{RADS}\, i\cap {csPCa}+\right)}{{{\, \#\, }}(\ge {PI}-{RADS\; i}\cap {path}\, +)}$$

$${estimated} \, \left({observed}\right) \, {NPV}=\frac{{estimated}\, \left({observed}\right){{\, \#\, \, }}\left({PI}-{RADS}\, 1-2\cap {csPCa}-\right)}{{{\, \#\, }}({PI}-{RADS}1-2\cap {path}\, +)}$$

$${estimated} \, \left({observed}\right) \, {CDR}=\frac{{estimated} \, \left({observed}\right){{\, \# \, }}\left({PI}-{RADS}\ge 3\cap {csPCa}+\right)}{{{\, \# \, }}({total})}$$

$${AIR}=\frac{{{\# \,}}({PI}-{RADS}\ge 3)}{{{\#}}\,({total})}$$

$${Pathological\; confirmation\; rate}=\frac{{{\#}}\, ({PI}-{RADS} \, i\cap {path}+)}{{{\#}}\, ({PI}-{RADS}i)}$$

$${Prevalence\; of\; csPCa}=\frac{{estimated}\, {{\#}}\, ({csPCa}+)}{{{\#}}\, ({total})}$$

$${Abbreviations}:{\#}\, {number\; of\; examinations};i=3,4,5;\\ {path}={prostate\; pathology\; within\; one\; year\; after\; the\; MRI}$$

The “observed” diagnostic performance metrics were defined for patients with pathological confirmation, whereas the “estimated” metrics were for patients with and without pathological confirmation, if appropriate. The CDR and AIR were calculated at PI-RADS ≥ 3. Note that the “estimated” items in a whole population, regardless of the presence of prostate pathology, were derived from the sum of the observed number of patients with csPCa in the population with pathology and the estimated number of patients with csPCa in the population without pathology.

For comparison with our estimated statistics, published studies that evaluated the diagnostic performance of prostate MRI were searched. Although most studies are for patients who have already been planned for prostate biopsy, we considered that two multi-center prospective studies evaluating the diagnostic performance of MRI-guided biopsy may potentially reflect the entire prostate MRI populations performed with clinical suspicion of csPCa [27, 28]. Van der Leest et al enrolled 626 biopsy-naive patients with clinical suspicion of csPCa and performed biopsies in all cases including 309 (49.3%) PI-RADS 1–2 examinations. Rouvier et al enrolled 251 biopsy-naive patients with clinical suspicion of csPCa and performed biopsies in all cases including 53 (21.1%) had PI-RADS 1–2 examinations. Since those studies focused on biopsy-naive patients, the above statistics in the current study were reported separately for all patients, biopsy-naive patients, and those with previous benign prostate biopsies. We considered AUC as a more suitable performance metric for comparison than other metrics because it is theoretically independent of disease prevalence and is invariant to shifts in PI-RADS assignments. The AUC between the current study cohort and published studies [27, 28] were compared but without statistical test due to the limited number of studies for comparison (*n* = 2).

The secondary analysis evaluated the association between the presence of pathological confirmation and age, PSAD, and the presence of previous benign biopsy for each PI-RADS score. The PI-RADS score-level breakdown of csPCa was shown using the Gleason grading system [29]. All analyses were performed at the exam level and summarized at each facility. Python 3.11 was used with an alpha level of 0.05.

## Results

### Study exam cohort

A total of 30,620 prostate MRI examinations were performed between 2018 and 2022. Of these, 12,191 examinations from 10,718 unique patients were enrolled (mean age, 65.7 years ± 8.4 [standard deviation]). Those examinations were interpreted by 41 board-certified abdominal radiologists and included 6761 PI-RADS 1–2 examinations (55.4%). Post-MRI prostate pathology was obtained in 5670 examinations (46.5%) within 1 year after the MRI, and csPCa was found in 3086 examinations (25.3%). Table 1 shows the extracted characteristics of patients with and without pathological confirmation. Table 2 shows the association between the included features and the presence of csPCa in patients with pathological confirmation. Age and PSAD were significantly higher in patients with csPCa than those without csPCa. The pathological confirmation rate was lowest in PI-RADS 1–2, ranging between 14.0 and 18.2% across facilities (Table 3).

**Table 1:** Patient characteristics

		Data			Number of missing values	
		With pathology (*n* = 5620)	Without pathology (*n* = 6521)	*p*-value	With pathology	Without pathology
Age (years)^a^		66.2 ± 8.0	65.3 ± 8.7	< 0.001		
PSA (ng/mL)^b^		6.8 (5.1, 9.8)	5.8 (4.1, 8.4)	< 0.001	560 (9.9%)	732 (11.2%)
Previous prostate biopsy	Naive	2147 (37.9%)	1960 (30.1%)	< 0.001		
	Benign	1272 (22.4%)	2356 (36.1%)			
	Unknown	2251 (39.7%)	2205 (33.8%)			
Family history of prostate cancer (+)		1723 (30.4%)	1667 (25.6%)	< 0.001		
Family history of breast cancer (+)		578 (10.2%)	701 (10.7%)	0.33		
Focal nodule on digital rectal exam (+)		546 (9.6%)	431 (6.6%)	< 0.001		
Race	Caucasian	5208 (91.9%)	5911 (90.6%)	< 0.001		
	African American	225 (4.0%)	229 (3.5%)			
	Asian	93 (1.6%)	144 (2.2%)			
	Others or unknown	144 (2.5%)	237 (3.6%)			
Facility	I	3027 (53.4%)	3479 (53.4%)	0.68		
	II	1190 (21.0%)	1405 (21.5%)			
	III	1453 (25.6%)	1637 (25.1%)			
PI-RADS	1–2	1041 (18.4%)	5720 (87.7%)	< 0.001		
	3	933 (16.5%)	415 (6.4%)			
	4	2052 (36.2%)	231 (3.5%)			
	5	1644 (29.0%)	155 (2.4%)			
Prostate volume (mL)^b^		46.8 (34.0, 66.6)	59.3 (41.0, 87.0)	< 0.001		
PSAD (ng/mL²)^b^		0.14 (0.10, 0.22)	0.09 (0.06, 0.13)	< 0.001		

Empty cells in the "Number of missing values" column represent no missing values. Unless otherwise specified, other data are the number of examinations, with percentages in parentheses. Proportions were compared using the chi-squared test

*PI-RADS* Prostate Imaging-Reporting and Data System, *PSA* prostate-specific antigen, *PSAD* prostate-specific antigen density

^a^ Data are the means, with the standard deviation in parentheses. The means were compared using the unpaired *t*-test

^b^ Data are the medians, with 1st and 3rd quartiles in parentheses. The medians were compared using the Wilcoxon rank sum test

**Table 2:** Association between the included predictive variables and the presence of clinically significant prostate cancer

		csPCa (+)	csPCa (−)	*p*-value	Number of missing values
PI-RADS	Variables				
1–2	Number of examinations	165 (15.9%)	876 (84.1%)		
	Age (years)^a^	64.5 ± 7.2	62.7 ± 7.3	0.003	0
	Benign biopsy history (+)	38 (23.0%)	263 (30.0%)	0.09	0
	Facility I	87 (52.7%)	407 (46.5%)	0.31	0
	Facility II	29 (17.6%)	187 (21.3%)		
	Facility III	49 (29.7%)	282 (32.2%)		
	PSA (ng/mL)^b^	6.7 (5.3, 9.3)	6.7 (4.9, 9.4)	0.56	141 (13.5%)
	Prostate volume (mL)^b^	43.9 (31.5, 64.5)	59.0 (43.9, 80.0)	< 0.001	45 (4.3%)
	PSAD (ng/mL²)^b^	0.16 (0.11, 0.21)	0.12 (0.08, 0.16)	< 0.001	163 (15.7%)
3	Number of examinations	284 (30.4%)	649 (69.6%)		
	Age (years)^a^	65.7 ± 7.0	63.0 ± 7.4	< 0.001	0
	Benign biopsy history (+)	50 (17.6%)	214 (33.0%)	< 0.001	0
	Facility I	214 (75.4%)	409 (63.0%)	< 0.001	0
	Facility II	24 (8.5%)	132 (20.3%)		
	Facility III	46 (16.2%)	108 (16.6%)		
	PSA (ng/mL)^b^	6.2 (5.0, 8.4)	6.2 (4.7, 8.5)	0.22	73 (7.8%)
	Prostate volume (mL)^b^	41.0 (32.0, 57.8)	56.8 (40.0, 79.0)	< 0.001	36 (3.9%)
	PSAD (ng/mL²)^b^	0.15 (0.11, 0.21)	0.11 (0.08, 0.15)	< 0.001	97 (10.4%)
4	Number of examinations	1271 (61.9%)	781 (38.1%)		
	Age (years)^a^	67.4 ± 7.4	64.9 ± 7.3	< 0.001	0
	Benign biopsy history (+)	188 (14.8%)	239 (30.6%)	< 0.001	0
	Facility I	685 (53.9%)	364 (46.6%)	< 0.001	0
	Facility II	215 (16.9%)	238 (30.5%)		
	Facility III	371 (29.2%)	179 (22.9%)		
	PSA (ng/mL)^b^	6.4 (5.0, 8.7)	5.8 (4.4, 8.4)	< 0.001	203 (9.9%)
	Prostate volume (mL)^b^	40.0 (30.6, 55.7)	52.0 (37.0, 72.3)	< 0.001	46 (2.2%)
	PSAD (ng/mL²)^b^	0.16 (0.11, 0.23)	0.11 (0.08, 0.17)	< 0.001	236 (11.5%)
5	Number of examinations	1366 (83.1%)	278 (16.9%)		
	Age (years)^a^	69.8 ± 8.0	67.0 ± 8.2	< 0.001	0
	Benign biopsy history (+)	194 (14.2%)	86 (30.9%)	< 0.001	0
	Facility I	756 (55.3%)	105 (37.8%)	< 0.001	0
	Facility II	267 (19.5%)	98 (35.3%)		
	Facility III	343 (25.1%)	75 (27.0%)		
	PSA (ng/mL)^b^	8.8 (6.1, 14.8)	6.7 (5.3, 10.6)	< 0.001	143 (8.7%)
	Prostate volume (mL)^b^	41.0 (32.2, 57.0)	51.0 (37.0, 73.0)	< 0.001	41 (2.5%)
	PSAD (ng/mL²)^b^	0.21 (0.14, 0.37)	0.15 (0.09, 0.22)	< 0.001	171 (10.4%)

Unless otherwise specified, data are the number of examinations with percentages. Parentheses enclose the percentages within the group with or without csPCa. Proportions were compared using the chi-squared test

*csPCa* clinically significant prostate cancer, *PI-RADS* Prostate Imaging-Reporting and Data System, *PSA* prostate-specific antigen, *PSAD* prostate-specific antigen density

^a^ Data are the means with standard deviations. The means were compared using the unpaired *t*-test

^b^ Data are the medians, with 1st and 3rd quartiles in parentheses. The medians were compared using the Wilcoxon rank sum test

**Table 3:** Facility-level pathological confirmation rates per PI-RADS score

PI-RADS	Facility I	Facility II	Facility III
1–2	14.0% (494/3530)	15.3% (216/1414)	18.2% (331/1817)
3	70.1% (623/889)	62.2% (156/251)	74.0% (154/208)
4	91.7% (1049/1144)	87.3% (453/519)	87.3% (453/519)
5	91.3% (861/943)	88.8% (365/411)	93.9% (418/445)

The numerators in parentheses are the number of examinations with post-MRI prostate pathology within 1 year after the MRI, while the denominators are the number of examinations with and without pathological confirmation

*PI-RADS* Prostate Imaging-Reporting and Data System

### Primary analysis

Table 4 shows the observed and estimated csPCa proportions per PI-RADS score. The estimation bias in patients with pathological confirmation ranged from −0.6% to 1.2%. The estimated csPCa proportion was lower than the observed proportion in the PI-RADS 1–2 population (12.6–15.3% vs. 13.4–17.6%, respectively). In contrast, the opposite was found in the PI-RADS 5 population (75.4–88.3% vs. 73.2–87.8%, respectively).

**Table 4:** Estimated and observed proportions of clinically significant prostate cancer

		Patients without pathological confirmation	Patients with pathological confirmation	
PI-RADS	Facility	Estimation	Observation	Estimation bias (estimation - observation in out of bag)
1–2	I	15.3% [12.2–18.5%]	17.6% (87/494)	−0.6% [−6.8 to 5.9%]
	II	12.6% [8.8–16.7%]	13.4% (29/216)	0.5% [−9.0 to 10.0%]
	III	13.5% [10.3–16.8%]	14.8% (49/331)	0.0% [−8.0 to 7.7%]
3	I	31.4% [27.7–34.9%]	34.3% (214/623)	−0.2% [−7.2 to 7.0%]
	II	18.4% [13.5–23.5%]	15.4% (24/156)	1.2% [−9.6 to 11.8%]
	III	28.6% [22.3–35.1%]	29.9% (46/154)	0.4% [−14.3 to 14.8%]
4	I	64.6% [61.8–67.2%]	65.3% (685/1049)	−0.0% [−5.9 to 5.5%]
	II	45.7% [41.8–49.8%]	47.5% (215/453)	0.6% [−8.2 to 9.9%]
	III	69.9% [66.2–73.6%]	67.5% (371/550)	−0.2% [−8.7 to 8.0%]
5	I	88.3% [86.4–90.2%]	87.8% (756/861)	−0.4% [−4.7 to 3.9%]
	II	75.4% [71.3–79.4%]	73.2% (267/365)	0.8% [−9.0 to 10.4%]
	III	86.1% [83.1–88.8%]	82.1% (343/418)	0.2% [−7.2 to 7.9%]

The estimated proportions of clinically significant prostate cancer (csPCa) are based on patients without pathological confirmation. The average of the model’s outputs is shown for each PI-RADS score, assuming a 100% pathological confirmation rate

The observed csPCa proportions are based on patients with pathological confirmation

The estimation biases represent the differences between the estimated and the observed csPCa proportions in patients with pathological confirmation

The 95% confidence intervals are shown in square brackets

*PI-RADS* Prostate Imaging-Reporting and Data System

**Fig. 3:**
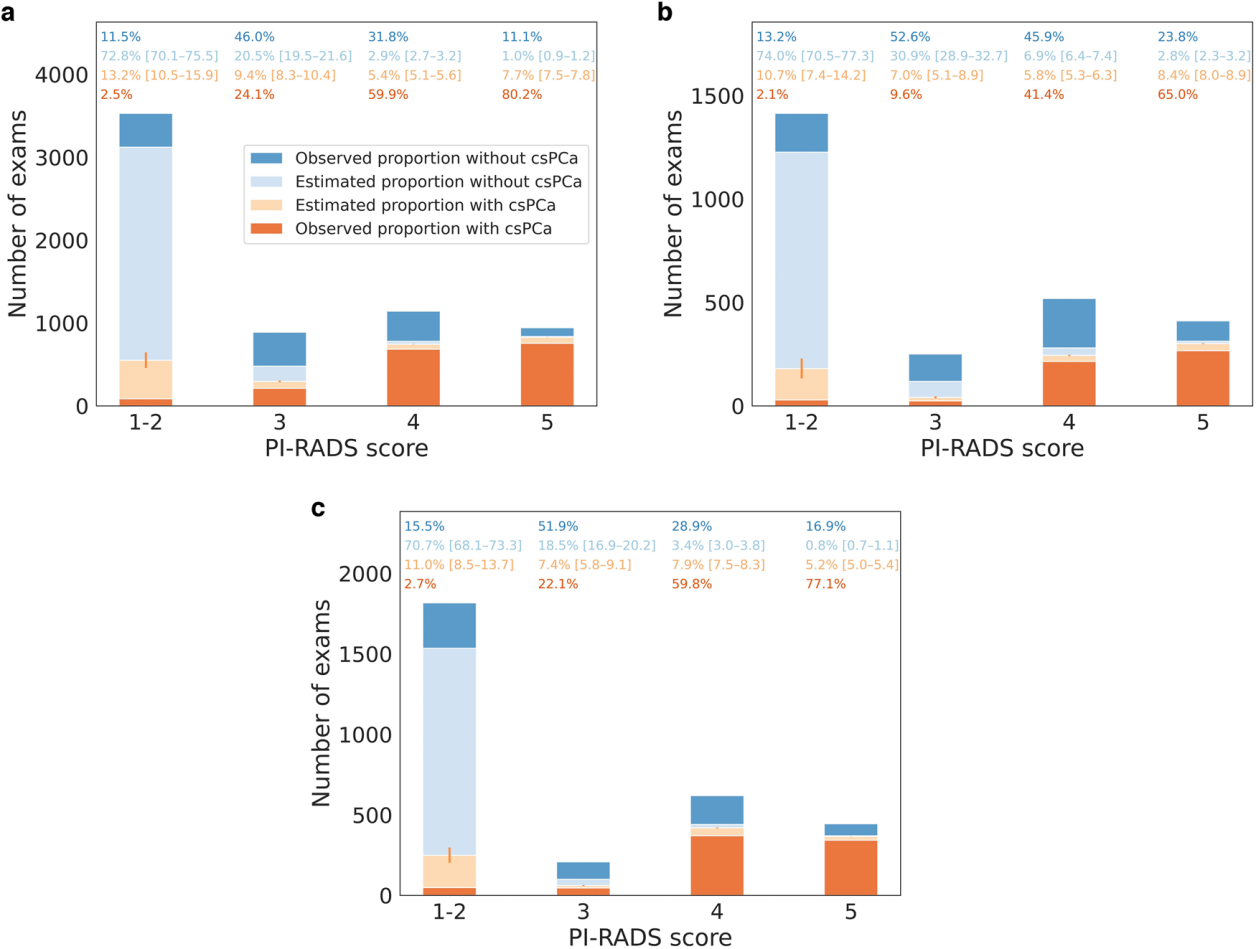
Observed and estimated proportions with clinically significant prostate cancer. **a** Facility I. **b** Facility II. **c** Facility III. The darker colors represent the examinations with pathological confirmation, while the lighter colors represent those without pathological confirmation. The numbers above the bars represent the PI-RADS score-level proportion of the categories corresponding to the identical color bar plots. The 95% confidence intervals of the estimated proportions are shown in square brackets. The vertical lines in light red represent the 95% confidence intervals of the estimated proportion of clinically significant prostate cancer. csPCa, clinically significant prostate cancer; PI-RADS, Prostate Imaging-Reporting and Data System

Figure 3 shows the bar plots representing the observed and estimated csPCa proportions. The estimated csPCa proportion was most uncertain in PI-RADS 1–2 (12.8–15.7%).

**Fig. 4:**
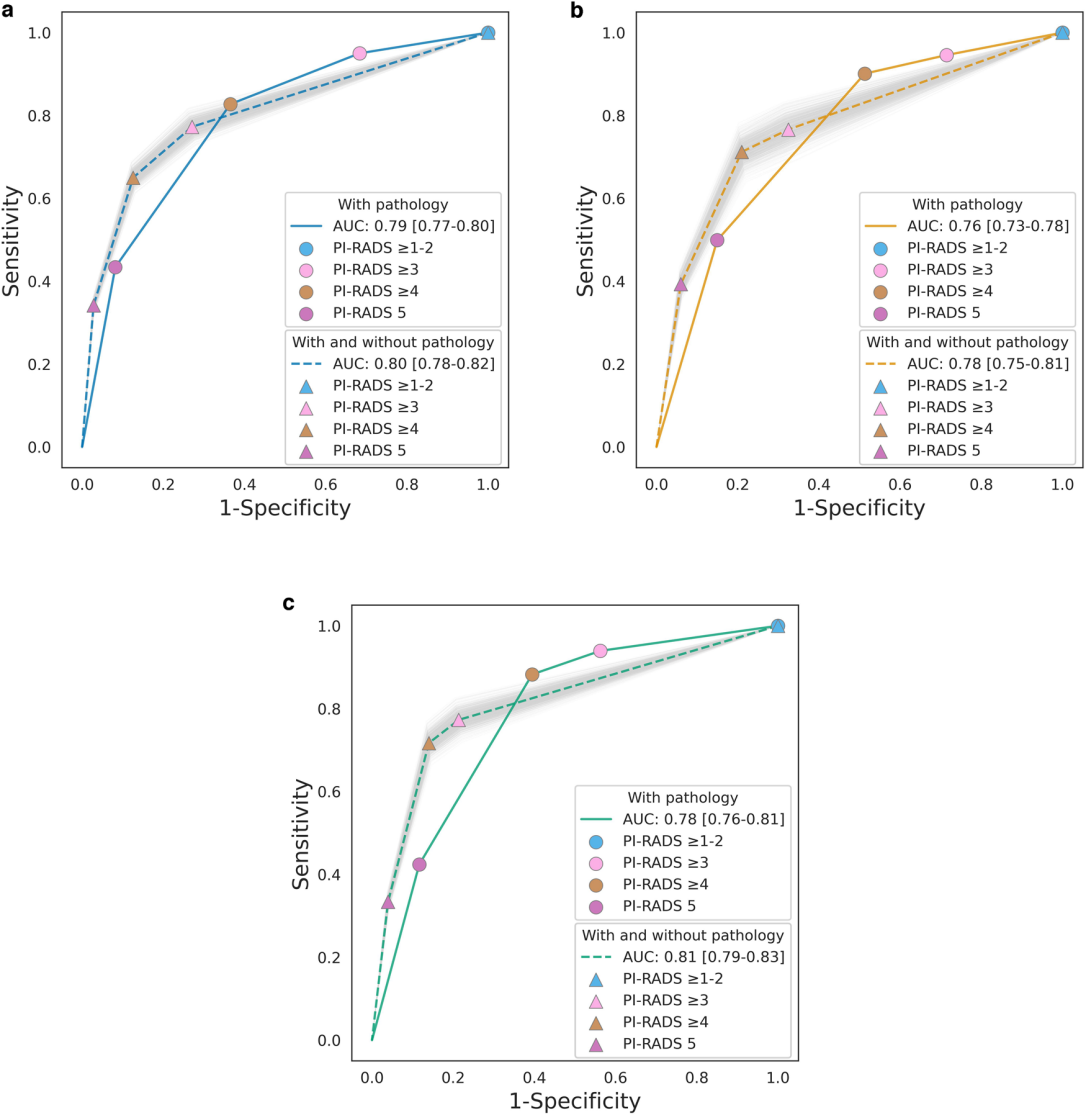
Observed and estimated receiver operating characteristic curves. **a** Facility I. **b** Facility II. **c** Facility III. The colored lines represent the receiver operating characteristic (ROC) curves in patients with pathological confirmation, while the same color dotted lines represent those in patients with and without pathological confirmation. Light gray areas represent the distribution of the ROC curves for each bootstrap repetition. The area under the ROC curve was shown with the 95% confidence interval in square brackets. The 95% confidence interval was calculated using the percentiles of the bootstrap distribution. AUC, area under the receiver operating characteristic curve; PI-RADS, Prostate Imaging-Reporting and Data System

Figure 4 shows the receiver operating characteristic curves of the PI-RADS score. The estimated AUC across facilities ranged from 0.78 to 0.81, while the observed AUC ranged from 0.76 to 0.79. Table 5 summarizes all PI-RADS performance metrics and csPCa prevalence at each facility. The estimated statistics were as follows (with observed statistics shown in parentheses, if appropriate): sensitivity at PI-RADS ≥ 3, 76.6–77.3%; specificity at PI-RADS ≥ 3, 67.5–78.6%; PPV at PI-RADS ≥ 3, 49.8–66.6% (52.0–66.7%); NPV, 84.4–87.2% (82.4–86.6%); CDR, 22.7–28.8% (19.5–25.4%); and csPCa prevalence, 29.6–37.7%. The largest inter-facility difference was seen in PPV at PI-RADS ≥ 3. Biopsy-naive patients had higher estimated sensitivity, PPV, CDR, AUC, and csPCa prevalence than those with previous benign biopsies in all facilities. In contrast, the estimated NPV in biopsy-naive patients was lower than in those with previous benign biopsies.

**Table 5:** Comparison of the diagnostic performance in biopsy-naive patients with published studies

		Current study			Published study	
	PI-RADS threshold	Facility I	Facility II	Facility III	van der Leest	Rouviere
Sensitivity	≥ 3	80.4% [77.8–83.2%]	79.5% [75.4–83.7%]	81.2% [78.3–84.3%]	93.2% (136/146)	93.6% (88/94)
	≥ 4	69.0% [66.8–71.3%]	73.5% [69.6–77.6%]	76.8% [74.1–79.6%]	89.0% (130/146)	83.0% (78/94)
	5	35.6% [34.5–36.8%]	39.5% [37.4–41.7%]	35.1% [33.9–36.4%]	65.8% (96/146)	58.5% (55/94)
Specificity	≥ 3	73.6% [72.9–74.3%]	68.3% [67.2–69.2%]	77.0% [76.3–77.6%]	62.3% (299/480)	29.9% (47/157)
	≥ 4	88.0% [87.6–88.3%]	78.4% [77.6–79.0%]	83.9% [83.5–84.3%]	69.4% (333/480)	61.8% (97/157)
	5	97.9% [97.8–98.0%]	93.8% [93.6–94.0%]	96.3% [96.1–96.4%]	90.6% (435/480)	93.0% (146/157)
PPV	≥ 3	70.9% [70.6–71.2%]	58.8% [58.2–59.4%]	71.4% [71.2–71.7%]	42.9% (136/317)	44.4% (88/198)
	≥ 4	82.1% [81.9–82.3%]	65.9% [65.7–66.3%]	77.2% [76.8–77.4%]	46.9% (130/277)	77.8% (98/126)
	5	93.2% [93.1–93.4%]	78.4% [77.7–79.0%]	87.0% [86.6–87.2%]	68.1% (96/141)	83.3% (55/66)
NPV		82.5% [79.5–85.4%]	85.4% [81.5–89.0%]	85.3% [82.4–88.1%]	96.8% (299/309)	88.7% (47/53)
CDR		35.7% [35.6–35.9%]	28.9% [28.6–29.1%]	33.6% [33.5–33.7%]	21.7% (136/626)	39.8% (137/344)
AIR		50.4% (1073/2130)	49.1% (421/858)	47.1% (527/1119)	50.6% (317/626)	78.9% (198/251)
Prevalence		44.4% [43.0–45.9%]	36.3% [34.4–38.4%]	41.4% [39.9–42.9%]	23.3% (146/626)	37.5% (94/251)
AUC		0.83 [0.81–0.84]	0.79 [0.76–0.82]	0.83 [0.81–0.85]	0.86 [0.83–0.89]	0.81 [0.76–0.86]

Performance metrics were calculated for the present study and two multi-center prospective studies that evaluated the diagnostic performance of MRI-guided biopsy (van der Leest: [28], Rouviere: [29])

The current study shows the estimated statistics calculated from all biopsy-naive patients with clinical suspicion of clinically significant prostate cancer (csPCa) in the three facilities. In contrast, the published studies show the observed statistics calculated from all enrolled biopsy-naive patients

When calculating the estimated statistics, the estimated number of examinations with csPCa was used, assuming a 100% pathological confirmation rate. The 95% confidence intervals are shown in square brackets

*AIR* abnormal interpretation rate, *CDR* cancer detection rate, *NPV* negative predictive value, *PI-RADS* Prostate Imaging-Reporting and Data System, *PPV* positive predictive value

Supplementary Table 1 compares the estimated diagnostic performance in biopsy-naive patients between the present study and the published studies [27, 28]. The estimated AUC was as follows: facility I–III, 0.79–0.83 vs. published studies, 0.81–0.86.

### Secondary analysis

Supplementary Table 2 shows the characteristics of the patients with and without pathological confirmation. Patients without pathological confirmation were significantly older, had lower PSAD values and a lower proportion of previous benign prostate biopsies than those with pathological confirmation. Supplementary Table 3 shows the PI-RADS score-level breakdown of csPCa. In 165 csPCa examinations with PI-RADS 1–2, 70.9% (117/165) and 20.6% (34/165) were ISUP Grades Group 2 and 3, respectively.

## Discussion

The current study collected common prostate cancer risk factors and developed risk prediction models to estimate the diagnostic performance of prostate MRI performed for clinical suspicion of csPCa. The sensitivity and specificity of PI-RADS ≥ 3 were estimated as 76.6–77.3% and 67.5–78.6%, respectively. The estimated NPV in the whole PI-RADS 1–2 population was 84.4–87.2%, which was 0.6–2.0% higher than the observed NPV in the PI-RADS 1–2 population with pathological confirmation.

The purpose of the current study was to understand the PI-RADS diagnostic performance in the entire population, including patients without pathological confirmation. It was assumed that the probability of harboring csPCa in patients without pathological confirmation could be adequately estimated using csPCa-associated clinical variables and data obtained from patients with pathological confirmation. This assumption is considered reasonable, especially in patients with pathological confirmation. Previous studies showed that the csPCa proportion can be reasonably estimated by using clinical variables [30–33]. The current study also showed a small estimation bias in the csPCa proportions, supporting this assumption.

However, these findings do not necessarily prove that the estimated csPCa proportion in patients without pathological confirmation is accurate. Population differences between those with and without pathological confirmation may exist, such as the documentation/extraction rate of the clinical information. For example, urologists might record csPCa-related clinical factors more frequently in patients who were undergoing prostate biopsy than those who were not. This study requires an assumption of no significant difference in the unextracted clinical variables between the two populations, which is the largest limitation of this study. To support our estimated result, we compared the AUCs in biopsy-naive patients to those in the previous prospective studies [27, 28]. Although we did not perform statistical tests due to the limited number of studies, the mean AUCs are close between the three facilities in the current study and the two published studies.

PI-RADS 1–2 accounted for more than 50% of prostate MRI examinations, but less than 20% of those examinations had pathological confirmation. Therefore, the estimated csPCa proportion in PI-RADS 1–2 substantially impacted the overall estimated statistics. By adding the estimated data, the receiver operating characteristic curve shifted to the lower left compared to that created from the observed data alone. The sensitivity decreased while the specificity increased in all PI-RADS score thresholds. The estimated sensitivity of PI-RADS ≥ 3 was about 77%, indicating that about 23% of csPCa was categorized as PI-RADS 1–2. Also, about 13–15% of the patients who were categorized PI-RADS 1–2 and without pathological confirmation were estimated to have csPCa. Possible reasons for false negatives include lesion characteristics such as small size and tumor location of the anterior region or transition zone [34–36]. Most of those missed csPCa may be intermediate-risk cancers (Grade Groups 2–3), given that over 90% of pathologically proven csPCa in PI-RADS 1–2 were in intermediate-risk groups. Another possible reason is decreased image quality. For example, a previous study [19] showed decreased CDR in patients with moderate to severe susceptibility artifacts from hip prostheses, mainly attributed to the increased frequency of PI-RADS 1–2. Adequate diagnostic image quality is essential for the efficient MRI-directed diagnostic pathway, and standardization of image quality metrics (PI-QUAL) has been proposed [37–39]. On the other hand, about 21–33% of non-csPCa cases are estimated to be categorized as PI-RADS ≥ 3. Possible reasons for false positives include inflammatory changes and benign hyperplasia [40]. As a previous study [4] reported, there was an inter-facility difference in PPV. Possible reasons behind this include differences in the distribution of age and race, radiologists’ threshold in assigning PI-RADS scores, and indications of prostate biopsies [8, 41].

This study revealed the diagnostic challenges of prostate MRI, especially in patients with previous benign prostate biopsies. Compared to biopsy-naive patients, the estimated sensitivity of PI-RADS ≥ 3 and AUC were lower by 12–17% and 0.18–0.21, respectively. One plausible explanation is that many easily identifiable csPCa have already been diagnosed in previous biopsies, making it relatively difficult to detect csPCa in the remaining population. Given the difference in diagnostic performance, it is preferable to calculate the performance metrics separately according to previous biopsy status.

In PI-RADS 1–2, patients without pathological confirmation had significantly lower PSAD values, a significantly higher proportion of a previous history of benign biopsy, and were significantly older than those with pathological confirmation. The former two factors indicated that prostate biopsy was not performed due to a relatively low risk of csPCa, while the last factor suggested that biopsy was avoided because of the lower clinical impact of diagnosing csPCa despite a higher csPCa risk. Overall, the estimated csPCa proportion was slightly lower in patients without pathological confirmation than in those with pathological confirmation. On the other hand, the estimated csPCa proportion was slightly higher in patients without pathological confirmation than in those with pathological confirmation in PI-RADS 5. This may indicate that urologists avoided prostate biopsy despite the high risk of csPCa based on patient conditions, such as comorbidities.

Our proposed method allows us to estimate several performance metrics, including sensitivity and specificity. Comparing these metrics among several institutions would be valuable feedback for improving diagnostic performance for each center. Also, these estimated metrics may be used to recognize facilities with high diagnostic performance, such as the American College of Radiology Prostate Cancer MRI Center Designation [42].

In addition to the potential biases mentioned above, the current study’s limitations include a potential selection bias due to the retrospective single-institution study design, although it consists of a multi-state health system. Also, this study assumed that there was no significant change in biopsy indications during the research period.

In summary, the estimated AUC, sensitivity at PI-RADS ≥ 3, and specificity at PI-RADS ≥ 3 were 0.78–0.81, 76.6–77.3% and 67.5–78.6%, respectively. The estimated statistics varied depending on the previous biopsy status. We expect the calculated statistics to help us understand the true PI-RADS performance and serve as a reference for future studies.

## Supplementary information

ELECTRONIC SUPPLEMENTARY MATERIAL

## Abbreviations

AIR: Abnormal interpretation rate
AUC: Area under the receiver operating characteristic curve
CDR: Cancer detection rate
csPCa: Clinically significant prostate cancer
NPV: Negative predictive value
PI-RADS: Prostate Imaging-Reporting and Data System
PPV: Positive predictive value
PSA: Prostate-specific antigen
PSAD: Prostate-specific antigen density
