# Supplementary material for: Estimated diagnostic performance of prostate MRI performed with clinical suspicion of prostate cancer
Source: Insights Imaging. 2024 Nov 15;15:271. doi: 10.1186/s13244-024-01845-y (PMC11568117; doi:10.1186/s13244-024-01845-y)
Supplement: Supplementary file 1 — ELECTRONIC SUPPLEMENTARY MATERIAL [file 13244_2024_1845_MOESM1_ESM.pdf]

Estimated diagnostic performance of prostate MRI performed with clinical suspicion of prostate cancer

ELECTRONIC SUPPLEMENTARY MATERIAL

Supplemental Table 1: Summary of PI-RADS diagnostic performance and estimated prevalence at each facility

	PI–RADS threshold	Facility	Observed (all)	Observed (negative)	Observed (naive)	Estimated	Estimated (negative)	Estimated (naive)
**Sensitivity**	**≥3**	I				77.3% [74.3–80.3%]	63.9% [58.0–69.9%]	80.4% [77.8–83.2%]
		II				76.6% [71.9–81.5%]	67.4% [59.6–75.2%]	79.5% [75.4–83.7%]
		III				77.3% [74.0–80.7%]	63.9% [56.8–71.3%]	81.2% [78.3–84.3%]
	**≥4**	I				65.0% [62.5–67.5%]	49.9% [45.2–54.6%]	69.0% [66.8–71.3%]
		II				71.2% [66.8–75.8%]	62.8% [55.6–70.1%]	73.5% [69.6–77.6%]
		III				71.7% [68.6–74.8%]	58.0% [51.6–64.5%]	76.8% [74.1–79.6%]
	**5**	I				34.2% [32.9–35.5%]	24.1% [21.9–26.4%]	35.6% [34.5–36.8%]
		II				39.3% [36.8–41.9%]	35.3% [31.3–39.3%]	39.5% [37.4–41.7%]
		III				33.4% [32.0–34.9%]	28.2% [25.0–31.6%]	35.1% [33.9–36.4%]

**Specificity**	**≥3**	I				73.0% [72.3–73.6%]	74.9% [74.2–75.6%]	73.6% [72.9–74.3%]
		II				67.5% [66.6–68.4%]	67.4% [66.5–68.2%]	68.3% [67.2–69.2%]
		III				78.6% [78.1–79.2%]	83.5% [82.9–84.0%]	77.0% [76.3–77.6%]
	**≥4**	I				87.4% [87.2–87.7%]	89.4% [89.0–89.6%]	88.0% [87.6–88.3%]
		II				79.0% [78.4–79.5%]	80.3% [79.7–80.8%]	78.4% [77.6–79.0%]
		III				86.0% [85.6–86.3%]	89.1% [88.7–89.5%]	83.9% [83.5–84.3%]
	**5**	I				97.2% [97.1–97.3%]	97.9% [97.9–98.0%]	97.9% [97.8–98.0%]
		II				94.0% [93.8–94.2%]	94.5% [94.3–94.7%]	93.8% [93.6–94.0%]
		III				96.0% [95.9–96.1%]	95.9% [95.7–96.1%]	96.3% [96.1–96.4%]
**PPV**	**≥3**	I	65.3% (1,655/2,533)	46.3% (253/546)	72.8% (692/951)	62.9% [62.6–63.2%]	43.2% [42.4–44.1%]	70.9% [70.6–71.2%]
		II	52.0% (506/974)	33.1% (82/248)	60.5% (216/357)	49.8% [49.3–50.3%]	30.9% [30.3–31.6%]	58.8% [58.2–59.4%]
		III	67.7% (760/1,122)	54.8% (97/177)	72.2% (346/479)	66.6% [66.2–66.9%]	52.0% [50.9–52.8%]	71.4% [71.2–71.7%]
	**≥4**	I	75.4% (1,441/1,910)	58.4% (213/365)	82.2% (610/742)	75.5% [75.3–75.6%]	58.3% [58.1–58.6%]	82.1% [81.9–82.3%]
		II	58.9% (482/818)	40.8% (78/191)	65.9% (205/311)	58.8% [58.5–59.1%]	40.8% [40.4–41.7%]	65.9% [65.7–66.3%]
		III	73.8% (714/968)	60.3% (91/151)	77.1% (330/428)	73.8% [73.5–74.1%]	59.9% [59.3–60.5%]	77.2% [76.8–77.4%]

	**5**	I	87.8% (756/861)	77.6% (104/134)	93.5% (314/336)	87.8% [87.7–88.0%]	77.6% [76.9–77.6%]	93.2% [93.1–93.4%]
		II	73.2% (267/365)	58.4% (45/77)	77.9% (109/140)	73.4% [73.0–74.0%]	58.1% [56.7–58.9%]	78.4% [77.7–79.0%]
		III	82.1% (343/418)	65.2% (45/69)	86.7% (156/180)	82.3% [82.0–82.5%]	65.8% [64.9–66.2%]	87.0% [86.6–87.2%]
**NPV**		I	82.4% (407/494)	85.3% (139/163)	81.3% (148/182)	84.4% [81.7–87.0%]	87.4% [83.9–90.4%]	82.5% [79.5–85.4%]
		II	86.6% (187/216)	93.8% (60/64)	83.1% (64/77)	87.2% [83.8–90.5%]	90.4% [86.8–93.5%]	85.4% [81.5–89.0%]
		III	85.2% (282/331)	86.5% (64/74)	86.1% (87/101)	86.3% [83.6–88.8%]	89.1% [85.5–92.3%]	85.3% [82.4–88.1%]
**CDR**		I	25.4% (1,655/6,506)	12.6% (253/2,005)	32.5% (692/2,130)	28.8% [28.6–28.9%]	14.7% [14.4–14.9%]	35.7% [35.6–35.9%]
		II	19.5% (506/2,595)	9.8% (82/833)	25.2% (216/858)	22.7% [22.5–22.9%]	12.0% [11.8–12.3%]	28.9% [28.6–29.1%]
		III	24.6% (760/3,090)	12.3% (97/790)	30.9% (346/1,119)	27.4% [27.3–27.6%]	14.0% [13.8–14.2%]	33.6% [33.5–33.7%]
**AIR**		I	45.7% (2,976/6,506)	34.0% (681/2,005)	50.4% (1,073/2,130)			
		II	45.5% (1,181/2,595)	38.8% (323/833)	49.1% (421/858)			
		III	41.2% (1,273/3,090)	26.8% (212/790)	47.1% (527/1,119)			
**Prevalence**		I				37.3% [35.8–38.7%]	23.0% [21.0–25.3%]	44.4% [43.0–45.9%]
		II				29.6% [27.8–31.5%]	17.9% [15.9–20.0%]	36.3% [34.4–38.4%]
		III				35.5% [34.0–37.1%]	21.9% [19.6–24.5%]	41.4% [39.9–42.9%]

**AUC**		I	0.79 [0.77–0.80]	0.76 [0.72–0.79]	0.82 [0.79–0.84]	0.80 [0.78–0.82]	0.73 [0.70–0.77]	0.83 [0.81–0.84]
		II	0.76 [0.73–0.78]	0.77 [0.72–0.82]	0.75 [0.70–0.79]	0.78 [0.75–0.81]	0.73 [0.68–0.77]	0.79 [0.76–0.82]
		III	0.78 [0.76–0.81]	0.74 [0.68–0.80]	0.79 [0.75–0.82]	0.81 [0.79–0.83]	0.75 [0.71–0.79]	0.83 [0.81–0.85]

The observed statistics were calculated from patients with pathological confirmation, whereas the estimated statistics were calculated from patients with and without pathological confirmation. The estimated number of examinations with clinically significant prostate cancer was used, assuming a 100% pathological confirmation. Statistics were reported separately for all patients, biopsy-naive patients, and those with previous benign prostate biopsies. The 95% confidence intervals are shown in square brackets.

AIR = abnormal interpretation rate, CDR = cancer detection rate, NPV = negative predictive value, PI-RADS = Prostate Imaging-Reporting and Data System, PPV = positive predictive value

Supplemental Table 2: Comparison between patients with and without pathological confirmation

PI-RADS	Facility	Variables	With pathological confirmation	Without pathological confirmation	P-Value
**1–2**	**I**	**Age (year)**	63.0 ± 7.3	64.8 ± 8.6	<0.001
		**PSAD (ng/ml²)**	0.12 (0.08, 0.17)	0.09 (0.06, 0.12)	<0.001
		**Benign biopsy history (+)**	33.0% (163/494)	38.2% (1161/3,036)	0.029
	**II**	**Age (year)**	61.9 ± 7.3	64.1 ± 8.1	<0.001
		**PSAD (ng/ml²)**	0.12 (0.09, 0.17)	0.09 (0.06–0.13)	<0.001
		**Benign biopsy history (+)**	29.6% (64/216)	37.2% (446/1,198)	0.039
	**III**	**Age (year)**	63.6 ± 7.3	65.6 ± 8.5	<0.001
		**PSAD (ng/ml²)**	0.12 (0.08, 0.17)	0.09 (0.07, 0.13)	<0.001
		**Benign biopsy history (+)**	22.4% (74/1486)	33.9% (504/1,486)	<0.001
**3**	**I**	**Age (year)**	63.6 ± 7.3	67.0 ± 9.0	<0.001
		**PSAD (ng/ml²)**	0.12 (0.09, 0.17)	0.10 (0.07, 0.16)	<0.001
		**Benign biopsy history (+)**	29.1% (181/623)	39.8% (106/266)	0.002
	**II**	**Age (year)**	62.6 ± 7.1	65.9 ± 8.1	0.001
		**PSAD (ng/ml²)**	0.11 (0.08, 0.17)	0.10 (0.07, 0.15)	0.09
		**Benign biopsy history (+)**	36.5% (57/156)	40.0% (38/95)	0.68
	**III**	**Age (year)**	66.1 ± 7.7	64.8 ± 8.6	0.35
		**PSAD (ng/ml²)**	0.11 (0.08, 0.17)	0.14 (0.08, 0.22)	0.048
		**Benign biopsy history (+)**	16.9% (26/154)	35.2% (19/54)	0.009
**4**	**I**	**Age (year)**	66.6 ± 7.4	70.2 ± 9.2	<0.001
		**PSAD (ng/ml²)**	0.14 (0.10, 0.21)	0.11 (0.07, 0.17)	0.001

		**Benign biopsy history (+)**	22.0% (231/1,049)	21.1% (20/95)	0.93
	**II**	**Age (year)**	65.1 ± 7.5	66.7 ± 9.7	0.19
		**PSAD (ng/ml²)**	0.12 (0.08, 0.19)	0.10 (0.07, 0.18)	0.26
		**Benign biopsy history (+)**	25.2% (114/453)	36.4% (24/66)	0.08
	**III**	**Age (year)**	67.2 ± 7.6	71.5 ± 9.3	<0.001
		**PSAD (ng/ml²)**	0.15 (0.10, 0.22)	0.12 (0.10, 0.18)	0.29
		**Benign biopsy history (+)**	14.9% (82/550)	15.7% (11/70)	1.00
**5**	**I**	**Age (year)**	69.4 ± 8.0	72.4 ± 9.0	0.005
		**PSAD (ng/ml²)**	0.21 (0.13, 0.35)	0.25 (0.14, 0.41)	0.38
		**Benign biopsy history (+)**	15.6% (134/861)	11.0% (9/82)	0.34
	**II**	**Age (year)**	68.1 ± 8.0	73.7 ± 9.7	<0.001
		**PSAD (ng/ml²)**	0.18 (0.12, 0.26)	0.21 (0.10, 0.36)	0.75
		**Benign biopsy history (+)**	21.1% (77/365)	28.3% (13/54)	0.36
	**III**	**Age (year)**	70.3 ± 8.3	76.0 ± 9.6	0.006
		**PSAD (ng/ml²)**	0.21 (0.14, 0.36)	0.31 (0.20, 0.42)	0.13
		**Benign biopsy history (+)**	16.5% (69/418)	18.5% (5/27)	0.79

The pathological confirmation rates within one year after the MRI are shown with breakdowns in parentheses. The mean ages are shown with standard deviations and were compared using the unpaired t-test. The median PSAD is shown with 1st and 3rd quartiles in parentheses and was compared using the Wilcoxon rank sum test. The percentages with a history of benign prostate biopsy are shown with breakdowns in parentheses and were compared using the chi-squared test.

PSAD = Prostate-specific antigen density

Supplemental Table 3: PI-RADS score-level breakdown of pathologically proven clinically significant prostate cancer

	PI-RADS 1-2 (n=165)	PI-RADS 3 (n=284)	PI-RADS 4 (n=1,271)	PI-RADS 5 (n=1,366)
**Grade group 2 (intermediate-risk)**	117 (70.9%)	203 (71.5%)	744 (58.5%)	467 (34.2%)
**Grade group 3 (high-risk)**	34 (20.6%)	51 (18.0%)	344 (27.1%)	362 (26.5%)
**Grade group 4 (high-risk)**	10 (6.1%)	12 (4.2%)	99 (7.8%)	202 (14.8%)
**Grade group 5 (high-risk)**	4 (2.4%)	18 (6.3%)	84 (6.6%)	328 (24.0%)
**Metastatic prostate cancer without detailed Gleason score**	0 (0.0%)	0 (0.0%)	0 (0.0%)	7 (0.5%)

The number of examinations is shown with percentages in parentheses. The grade group and risk categorization were based on the guidelines from the European Association of Urology [S1]. PI-RADS = Prostate Imaging-Reporting and Data System

[S1] Mottet N, van den Bergh RCN, Briers E, et al (2021) EAU-EANM-ESTRO-ESUR-SIOG Guidelines on Prostate Cancer-2020 Update. Part 1: Screening, Diagnosis, and Local Treatment with Curative Intent. Eur Urol 79:243–262.
